# High‐grade serous peritoneal cancer follows a high stromal response signature and shows worse outcome than ovarian cancer

**DOI:** 10.1002/1878-0261.12811

**Published:** 2020-10-23

**Authors:** Francis Jacob, Rosa Lina Marchetti, André B. Kind, Kenneth Russell, Andreas Schoetzau, Viola A. Heinzelmann‐Schwarz

**Affiliations:** ^1^ Ovarian Cancer Research Department of Biomedicine University Hospital Basel University of Basel Switzerland; ^2^ Department of Gynecology and Gynecological Oncology Hospital for Women University Hospital Basel Switzerland; ^3^ CARIS Life Sciences International Basel Switzerland

**Keywords:** gene signature, metastasis, ovarian cancer, peritoneal cancer, predictive biomarker

## Abstract

In the era of personalized medicine, where transition from organ‐based to individualized genetic diagnosis takes place, the tailoring of treatment in cancer becomes increasingly important. This is particularly true for high‐grade, advanced FIGO stage serous adenocarcinomas of the ovary (OC), fallopian tube (TC), and peritoneum (PC), which are currently all treated identically. We analyzed three independent patient cohorts using histopathologically classified diagnosis and various molecular approaches (transcriptomics, immunohistochemistry, next‐generation sequencing, fluorescent and chromogenic *in situ* hybridization). Using multivariate Cox regression model, we found that PC is more aggressive compared with advanced‐stage OC independent of residual disease as shown by an earlier relapse‐free survival in two large cohorts (HR: 2.63, CI: 1.59–4.37, *P* < 0.001, and HR: 1.66, CI: 1.04–2.63, *P* < 0.033). In line with these findings, transcriptomic data revealed differentially expressed gene signatures identifying PC as high stromal response tumors. The third independent cohort (*n* = 4054) showed a distinction between these cancer types for markers suggested to be predictive for chemotherapy drug response. Our findings add additional evidence that ovarian and peritoneal cancers are epidemiologically and molecularly distinct diseases. Moreover, our data also suggest consideration of the tumor‐sampling site for future diagnosis and treatment decisions.

## Graphical abstract

Serous peritoneal and high‐grade serous ovarian/tubal cancers are treated identically with maximal cytoreductive surgery and platinum‐based chemotherapy. Here, we provide epidemiological, clinical, and molecular evidence that peritoneal cancer is more aggressive and shows a distinct molecular signature on gene and protein levels. Our data suggest alternative treatment decisions and to consider the tumor‐sampling site for diagnosis in the future.

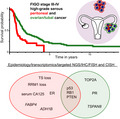

**Abbreviations**
AUC: area under the curve
CISH: chromogenic *in situ* hybridization
FISH: fluorescence *in situ* hybridization,
GLM: generalized linear model
LDA: linear discriminant analysis
NGS: next‐generation sequencing
OC: ovarian cancer
PC: peritoneal cancer
ROC: receiver operating characteristics
TC: tubal cancer

## 1 Introduction

High‐grade serous adenocarcinomas are typically *TP53* mutation‐harboring cancers, classified as primary ovarian (OC), fallopian tube (TC), or peritoneal (PC) carcinomas. Despite similarities in pathological appearance, clinical behavior, and prognosis [1], the degree to which these cancers differ is relatively unknown, and thus, they are treated identically with maximal cytoreductive surgery and platinum‐based chemotherapy. Recently, PARP inhibitors have been added as maintenance therapies [2, 3]. Niraparib monotherapy is currently being studied in patients with high‐grade serous adenocarcinomas treated with at least three prior chemotherapy regimens [4].

To date, the debate as to whether all three cancers are variants of the same malignancy or develop through different pathways remains controversial. It is particularly difficult to classify the disease when the primary site of origin cannot be precisely determined. Similarly, it is unclear whether the different phenotype occurs based on the origin or the site of development of the individual cancer. Nevertheless, the clinical benefits of distinguishing between these diseases are evident, as shown previously in the case of TC, which has been linked to frequent *BRCA* mutations [5, 6, 7]. The high risk of developing cancer supports pre‐menopausal prophylactic salpingo‐oophorectomy in women with these mutations. Once a tumor has developed, targeted therapy with PARP inhibitors is the new standard [8]. While widely regarded to be the same disease, recent findings suggest that PC and OC may be linked to different oncogenic pathways [9]. In addition, two epidemiological studies identified differences in risk patterns among these cancers, revealing that PC can be distinguished based on age, later menarche, and higher BMI [10, 11]. All three diseases share many histopathological and clinical characteristics, although OC is supposed to arise from the ovarian surface epithelium [12] and the fallopian tube [13], TC from the fimbrial end of the fallopian tube epithelium [14, 15], and PC presumably from the peritoneal mesothelium [16].

Despite shared histopathological features between primary PC and epithelial OC or TC [17], the following criteria were developed to define PC from the other two types: (a) Both ovaries are normal in size or enlarged by a benign process; (b) the involvement in extraovarian sites is greater than the involvement on the surface of the ovaries; (c) the ovaries are microscopically tumor‐free or exhibit only serosal or cortical invasions with dimensions smaller than 5 × 5 mm; and (d) histopathological and cytological characteristics of the tumor are predominantly of the serous histotype [18, 19]. These purely histopathological observations could be seen as imprecise, but highlight the underlying challenges and difficulties in identifying the site of origin at the time of clinical diagnosis, mainly due to the widespread dissemination and lack of reliable biomarkers. Discriminating between these cancers at an early stage is important, not just to facilitate the development of personalized treatment, but also to offer the possibility of bilateral oophorectomy/salpingectomy and/or removal of other organs or structures not yet identified as primary prevention for high‐risk patients.

Indeed, regarding personalized medicine, three studies have investigated high‐grade serous cancer (HGSC) cohorts for their molecular profile in order to distinguish different signatures, which could aid in developing specific treatments for PC, OC, and TC. In the first study by Tothill *et al*., serous and endometrioid ovarian cancers were distinguished by their molecular signature into high stromal response (C1), high immune response (C2), low‐malignant potential (C3), low stromal response (C4), mesenchymal (low immune signature, C5), and low‐grade endometrioid (C6). Here, the high stromal group C1 and mesenchymal C5 subtypes were classified as having the worst survival outcome [20]. Based on this signature and work by the Cancer Genome Atlas Research (TCGA) Network, Konecny *et al*. [21] used this HGSC cohort and separated them with regard to their different survival rate into an immunoreactive (C1), differentiated (C2), proliferative (C4), and mesenchymal (C5) phenotype. The clinical importance of this can be seen in a follow‐up paper where the classification of Konecny was applied for a subgroup of the ICON7 phase III study dataset, identifying a subgroup of patients with a specific gene subtype which may particularly benefit from bevacizumab [22]. In addition to and on another molecular level, we have previously used mass spectrometry to identify individual glycoprotein structures which could highly significantly discriminate OC and TC from PC [23].

In this publication, we aimed to identify potential clinical and molecular differences between OC and PC using three independent large datasets comprising of transcriptomics, next‐generation sequencing (NGS), and immunohistochemistry (IHC) data. So far, none has attempted to correlate the molecularly described discriminative patterns to the presently used clinical disease classifications. In the pursuit of defining a distinction between high‐grade serous ovarian and peritoneal cancers, we present here the first multicohort approach that combines epidemiology with a wide variety of molecular signatures to elucidate the distinctive properties of these cancer types for the subsequent development of targeted therapies.

## 2 Materials and methods

We analyzed four independent patient cohorts in order to determine whether OC, TC, and PC are distinct malignant diseases (Table 1 and described in detail below). All specimens used in this study have been diagnosed as either OC or PC based on their corresponding histopathological features. However, in some cases the diagnosis did not correspond to the location from which the tissue was derived, for example, an OC specimen was obtained from an omental metastasis and not from their presumed primary cancer location. For the purpose of this study, we performed two analyses: firstly, based on clinical diagnosis (e.g., OC); and secondly, on the location from where the tumor has been sampled.

**Table 1:** Summary of cohorts investigated in this study: ovarian cancer (OC), peritoneal cancer (PC), tubal cancer (TC), next‐generation sequencing (NGS), immunohistochemistry (IHC), fluorescence *in situ* hybridization (FISH), and chromogenic *in situ* hybridization (CISH).

Study	Country	Number of patients			Parameters evaluated	Comments/reference
		OC	PC	TC		
1	Switzerland	284	78		Histological and clinical characteristics and CA125 in kU·L^−1^	University Hospital Basel, Basel, Switzerland
2	Australia	199	33		Histological and clinical characteristics and gene expression data	Tothill *et al*. [20], GSE9899
3	various	3286	369	399	Histological characteristics, NGS, IHC, FISH, CISH	Caris Life Sciences, NGS performed since 01/2013
4	various	2970^a^			Gene expression for *ADH1B*, *FABP4*, and *TSPAN8* in ovarian cancer	‘curatedOvarianData’ package [24]

^a^ Manually curated data collection for gene expression meta‐analysis of patients with ovarian cancer obtained from 23 studies with curated and documented clinical metadata.

### 2.1 Cohort 1—Swiss cohort from the University Hospital Basel, Basel, Switzerland (clinicopathological data only)

In order to investigate the association between clinical diagnosis with disease recurrence and disease‐specific survival, we initially analyzed a retrospectively collected Swiss cohort consisting of 362 patients from the University Hospital Basel, Basel, Switzerland, from 1990 to 2018. This set was comprised of 284 and 78 cases diagnosed with OC and PC, respectively. Full clinicopathological information was available for this cohort.

### 2.2 Cohort 2—Australian cohort for transcriptomics published in Tothill *et al*. (2008) (clinicopathological and transcriptomic data)

Publicly available transcriptomic data were searched for cohorts comprising OC and PC with sufficient clinicopathological information. For the exploratory genetic analyses, we found one large publicly available Affymetrix oligonucleotide array dataset profiling patients with OC and PC that provided associated clinical information, including relapse‐ and disease‐specific survival. We used the given genetic profile and applied it to distinguish between the OC and the PC cohort. As most datasets did not pass the selection criteria of including both OC and PC, the Tothill analysis was the only one which fulfilled these distinction criteria [20]. CEL files (Affymetrix U133) and clinical data were downloaded from the Gene Expression Omnibus (GSE9899). In addition, the tumor location (cancer sampling site) for sampling was provided in the supplementary data of the original publication and this information was incorporated into our study [20]. We included only high‐grade serous OC and PC with advanced FIGO stages into our analysis to provide a homogeneous cancer group.

### 2.3 Cohort 3—US American cohort from Caris Life Sciences (minimal clinicopathological data and comprehensive molecular pathological data)

The clinical details and histological diagnosis of high‐grade serous cancers were based on the information provided by referring physicians. Four thousand and fifty‐four tumor patients were reported to have high‐grade serous cancer with advanced FIGO stage (III/IV) comprising TC (*n* = 399), OC (*n* = 3286), and PC (*n* = 369). The accompanying original pathology report did not provide data on FIGO stage, site of tumor sampling, disease recurrence, or prior treatments. The tissue samples were formalin‐fixed and paraffin‐embedded and obtained from the primary tumor or metastasis either at initial diagnosis or at disease recurrence. The Western Institutional Review Board, the IRB for Caris Life Sciences, deemed the study exempt from additional patient consent as it used previously collected de‐identified data. Specific testing was performed per physician request and included a combination Sanger or next‐generation sequencing (NGS) for identification of gene mutations, protein expression by standard immunohistochemistry (IHC), and investigation of gene amplifications by fluorescent *in situ* hybridization (FISH) or chromogenic *in situ* hybridization (CISH). The type of analyses performed and the specific biomarkers tested depended on the amount of tissue sample provided. The panel of tests evolved over time as new biomarker information was published. NGS was introduced in January 2013, and therefore, only limited number of tumor samples was analyzed by this method.

### 2.4 Statistical analysis

The ‘limma’ package from Bioconductor open‐source software for bioinformatics (r statistical software [24]) was used to identify differentially expressed genes between study groups. *P*‐values were adjusted by the Benjamini–Hochberg false discovery rate (FDR) method.

In order to perform stable feature selections for classification and prediction of subgroups, two popular methods were selected: random forest (RF) and penalized generalized linear model (pGLM).

RF was performed using the package ‘randomForestSRC’. Penalized GLM was performed using the package ‘glmnet’ within the r software. The stability of selected features was ensured by applying two algorithms. Subsequent ROC curves with corresponding AUC were estimated using the r package ‘pROC’. Additional linear discriminant analysis (LDA) and multivariate ANOVA were performed to test and visualize the performance of the feature selections (R package ‘lda’, ‘manova’). Survival analysis was conducted using Kaplan–Meier curves with corresponding log‐rank tests. Additionally, multivariate time‐to‐event analysis was done using Cox regression. Results are reported as hazard ratios (HR) with corresponding 95% confidence intervals and *P*‐values. (R package ‘Survival’). A *P*‐value < 0.05 was considered significant but interpreted exploratory. All evaluations were done using R version 3.1.3 and Bioconductor.

The ‘curatedOvarianData’ package was accessed as a comprehensive resource of gene expression in large ovarian cancer transcriptomic datasets (Table 1, cohort 4) to test selected genes and validate ovarian cancer prognostic models [25]. Statistical analysis of hazard ratio (HR) in the forest plot was obtained through the use of the software R following instruction from the ‘curatedOvarianData’ package [25].

## 3 Results

### 3.1 Patients with peritoneal cancer tend to have a higher age at diagnosis and show earlier relapse in two independent cohorts

**Fig. 1:**
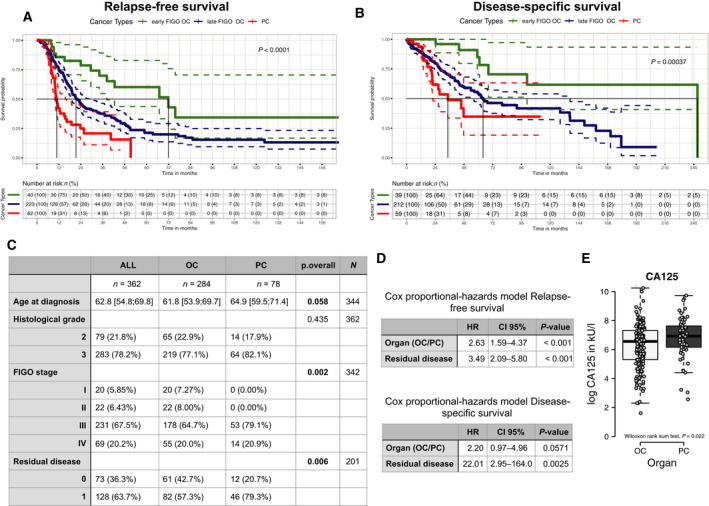
PC is more aggressive than advanced‐stage PC in the Swiss cohort. Kaplan–Meier survival curves of high‐grade serous cancer of the ovary (OC FIGO I/II, green, and OC FIGO III/IV, blue) and peritoneum (PC, red). Number of patients and percentage, legend, and level of significance (log‐rank test) are provided along within each Kaplan–Meier plot. (A) Relapse‐free survival (RFS) and (B) disease‐specific survival (DSS). (C) Descriptive statistics of histopathologically classified diagnosis of the Swiss cohort. (D) Cox proportional hazard model for RFS and DSS revealed organ (OC *versus* PC both FIGO stage III/IV) and residual disease as independent predictors, whereas remaining histopathologically parameters were not significant. (E) Box plot shows distribution of CA125 levels in kU/L prior surgery and evaluation using nonparametric test.

**Fig. 2:**
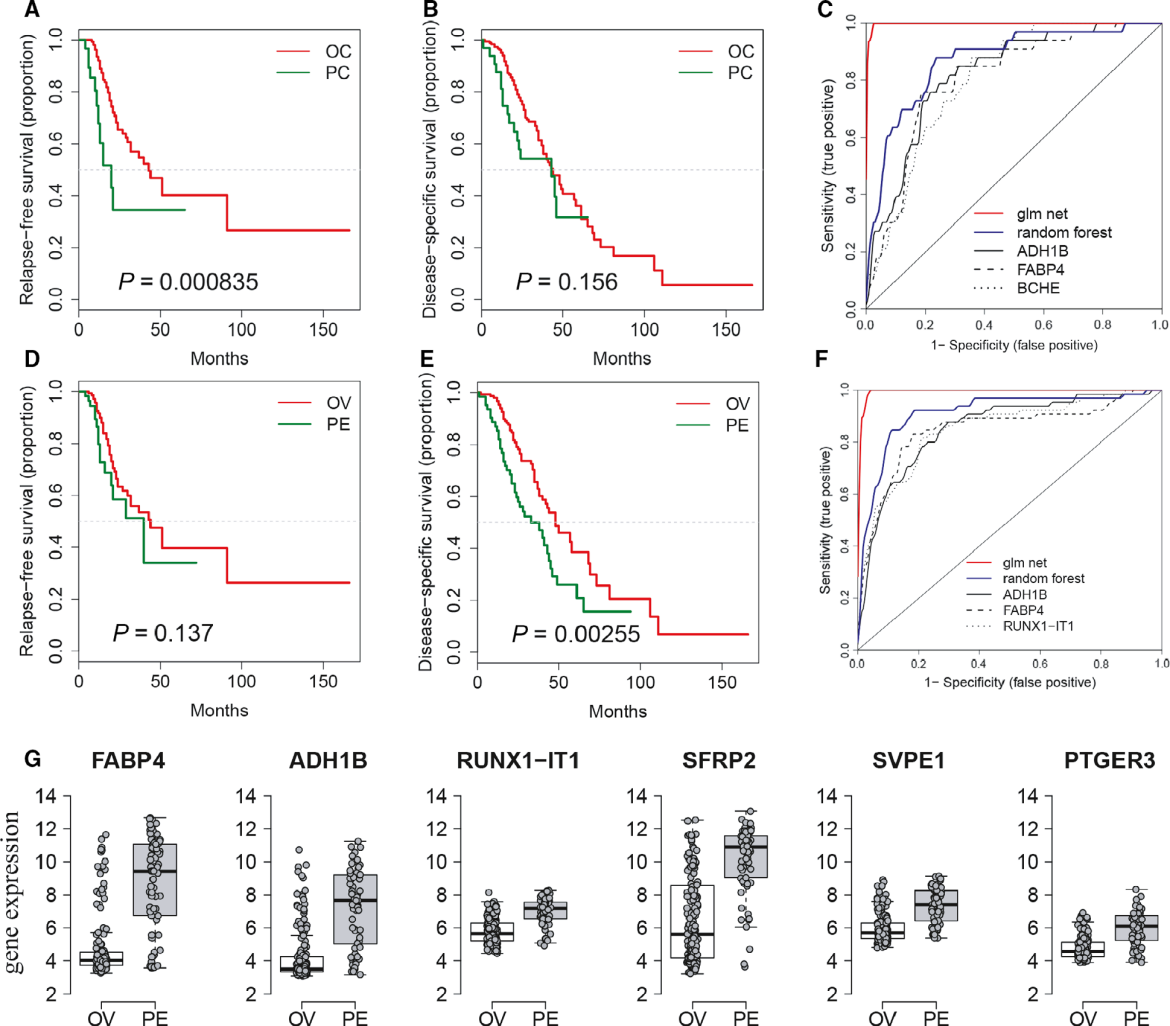
Differentially expressed genes distinguish OC and PC in cohort 2. (A) Kaplan–Meier curve estimates the relapse–free survival according to the diagnosed OC (OC, red) and PC (PC, green) cancer. (B) Kaplan–Meier curve for disease‐specific survival in diagnosed cancer patients. (C) ROC curves based on diagnosed ovarian and peritoneal cancer for *ADH1B* (AUC: 0.822, sensitivity: 81.8%, and specificity: 76.4%), *FABP4*, *BCHE*, and GLM‐ and RF‐ selected genes. (D) Kaplan–Meier curve estimates the relapse‐free survival according to the location, cancer tissue obtained from the ovary (OV) or peritoneum (PE). (E) Kaplan–Meier curve for disease‐specific survival comparing tumor location ovary and peritoneum. (F) ROC curve comparing ovary and peritoneum *ADH1B*, *FABP4*, *RUNX1‐IT1*, and GLM (*n* = 44)‐ and RF (*n* = 9)‐selected and linear combined genes (colors explained in the plot legend). (G) Box plot of most differentially ranked genes (*FABP4*, *ADH1B*, *RUNX‐IT1*, *SFRP2*, *SVPEI,* and *PTGER3*) comparing cancer tissue origins ovary (OV) and peritoneum (PE) in the HGSC cohort.

Patients with PC (median: 64.9 years, IQR: 59.9–71.4 years) appeared to be diagnosed at an older age (*P* = 0.058) than OC (median: 61.8 years, IQR: 53.9–69.7 years), which was in agreement with the results of previous studies [10, 26, 27]. Here, in cohort 1, which was comprised of 78.2% poorly differentiated and 21.8% moderately differentiated tumors of the ovary and peritoneum with 87.7% diagnosed at advanced FIGO (III/IV) stages, patients with PC showed a shorter relapse‐free survival (chi‐square 6.7, *P* = 0.009) and a trend toward a shorter disease‐specific survival (chi‐square 2.2, *P* = 0.1) in the entire cohort (Figure 1A,B). There was an overall significant difference for FIGO stage and residual disease (Figure 1C). With regard to relapse‐free survival, the Cox proportional hazard model identified residual disease and diagnosis as independent predictors (Figure 1D). Of note, patients with PC revealed significantly elevated CA125 serum levels as compared with OC (Figure 1E). The poor outcome observed in patients with PC was confirmed in cohort 2, from which 232 patients met the criteria for inclusion in our analysis. The dataset consists of 199 clinically diagnosed OC and 33 PC patients. As seen with cohort 1, PC relapsed significantly earlier than OC (chi‐square 11.2, *P* < 0.01, Figure 2A), while the disease‐specific survival showed a similar trend in both cancers (chi‐square 2.0, *P* > 0.05, Figure 2B). Again, multivariate Cox regression model independently predicted poor relapse‐free survival in PC compared with advanced FIGO stage OC (HR: 1.66, CI: 1.04–2.63, *P* = 0.033) together with residual disease (HR: 2.32, CI: 1.45–3.72, *P* < 0.001). A similar trend in age at diagnosis was also observed in cohort 2 comparing PC (median: 63.0 years, IQR: 58.0–70.0 years) with OC (median: 59.0 years, IQR: 53.0–70.0 years, *P* = 0.056).

### 3.2 Distinct gene expression signatures define OC and PC

In addition to our epidemiological findings, which are in line with those in the literature, we next asked whether gene expression signatures distinguish both cancer types. Regarding diagnosis, a total of 697 genes were identified to be differentially expressed in both cancer types (adjusted *P* < 0.05; Table S1, full list of genes available in Table S2) with alcohol dehydrogenase 1B (*ADH1B*) and fatty acid‐binding protein 4 (*FABP4*) being significantly elevated in PC. Gene combinations improved diagnostic performance of individual top candidate genes in case of random forest (RF)‐selected genes (*n* = 8) with an AUC of 0.870 (sensitivity: 87.9%; specificity: 77.9%). In contrast, penalized generalized linear model (pGLM) selected 40 genes clearly increasing the AUC to 0.998 (sensitivity: 100%; specificity: 98.5%; Figure 2C).

Apart from the analysis of clinicopathological characteristics, the cohort 2 also contains information regarding the sampling site (ovary *n* = 161 and peritoneum *n* = 65). Considering that the clinical outcome could depend on site as previously suggested based on cell surface glycan signatures using mass spectrometry [23], tumors collected from the peritoneum showed a trend toward earlier relapse (Figure 2D) and earlier time of death from disease (chi‐square 9.2, *P* < 0.01, Figure 2E). Comparing the sampling sites of HGSCs, we identified a higher number of differentially expressed genes (*n* = 2377) between the different diagnoses (Table S1, full list of genes available in Table S3). The feature selection algorithms revealed eight and 44 selected genes for RF and pGLM, respectively. Comparing the top discriminatory (*ADH1B*, *FABP4*, and *RUNX‐IT1*) genes with feature‐selected genes (RF and pGLM), the diagnostic performance was best for GLM (*n* = 44, AUC: 0.998, sensitivity: 100%, and specificity: 98.5%, Figure 2F). Interestingly, *ADH1B* and *FABP4* expression was significantly elevated in tumors localized in specimens originating from the peritoneum (Figure 2G).

### 3.3 PC show mostly high stromal and immune response profiles

**Fig. 3:**
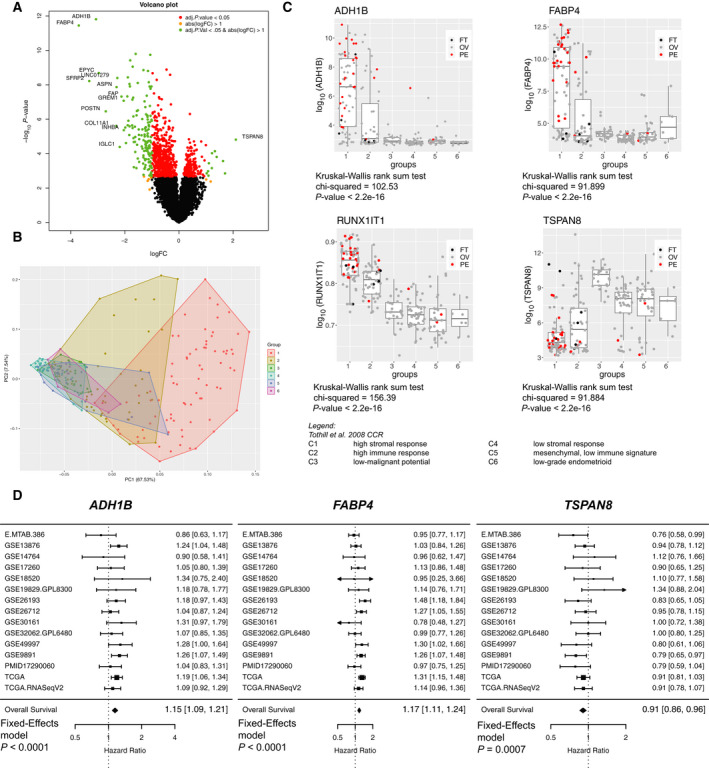
The gene signature of high stromal response ovarian cancer is reflected in the majority of PC. (A) Volcano plot showing the –log10 *P*‐values in relation to log2 fold change for the comparison ovarian *versus* peritoneal cancer. Colored points highlight genes according to adjusted parameters as described in the top right legend. (B) Principal component analysis (PCA) for top 60 genes (identified by limma) from the comparison ovarian *versus* peritoneal cancer highlighting the molecular groups defined in Tothill *et al*. 2008. Groups C1‐C6 are highlighted in different colors. (C) Box plot showing expression of selected candidate genes discriminating ovarian from peritoneal cancer for distribution over molecular groups C1 to C6. Corresponding statistics are provided along with each box plot. Diagnosed cancer types are highlighted in different colors. (D) The ‘curratedOvarianData’ database confirms *ADH1B* and *FABP4* as prognostic markers in patients with ovarian cancer. Forest plot of selected genes as a univariate predictor of overall survival using multiple transcriptomic datasets (annotated with study‐specific ID). Hazard ratio (HR) indicates the factor by which overall risk of death increases with a one standard deviation increase in expression for *ADH1B*, *FABP4*, and *TSPAN8*.

PC can be differentiated from ovarian cancer based on epidemiological, clinical, and molecular parameters. The molecular subgroup C1 (high stromal response) as published in the Tothill dataset corresponded to PC when we re‐analyzed this dataset by site of sampling (70.6%). Thus, we asked whether genes found to be significantly altered in PC as compared to OC are associated with described molecular groupings in this ovarian cancer cohort [20]. Here, genes identified from our comparison were further applied for a principal component analysis (Figure 3A,B). Again, this analysis revealed that PC fit the molecular signature of C1 (high stromal response) and also in C2 (high immune signature; Figure 3B). By selecting the genes *ADH1B*, *FABP4*, and *RUNX1‐IT1* (overexpressed in PC) which best discriminate OC and PC, the highest expression was observed in group C1 followed by C2. In contrast, *TSPAN8* (up in OC) showed significantly lower expression in C1 and C2 (Figure 3C). Importantly, from the patients diagnosed with PC, 79.3% belong to group C1. In addition to gene expression discriminating PC from OC, the pooled hazard ratio (HR) model in the transcriptomic curatedOvarianData set (*n* = 2970) identified *FABP4* (HR: 1.17) and *ADH1B* (HR: 1.15) as predictors for poor overall survival (HR: 1.16–1.20; Figure 3D). These results clearly demonstrate that PC follow gene signatures of highly aggressive tumors categorized into high stromal (C1) and immune response (C2), both of which have been described as the most aggressive ovarian cancer subtypes.

### 3.4 Prediction of therapeutic targets for OC and PC

The differences between OC and PC triggered us to investigate whether different treatment regimens should be considered for these molecularly different diseases. For this, we investigated cohort 3 at the time of their diagnosis. Caris Life Sciences has focused on analyzing biomarkers associated with clinical response to particular drug therapies. Data from NGS, FISH/CISH, and IHC were analyzed in this cohort. We found that all HGSCs showed a nearly identical mutational frequency for *TP53* (total: 79.0%; OC: 78.7%; PC: 78.5%; and TC: 81.0%). No other discriminating targetable mutation (*n* = 44) was found for any of the three cancer types using NGS. Interestingly, the mutation rates for commonly reported genes in ovarian cancer such as *RB1*, *PTEN*, *PIK3CA*, *NRAS*, *KRAS*, and *BRAF* were below 3% in the entire cohort (Table S4).

**Fig. 4:**
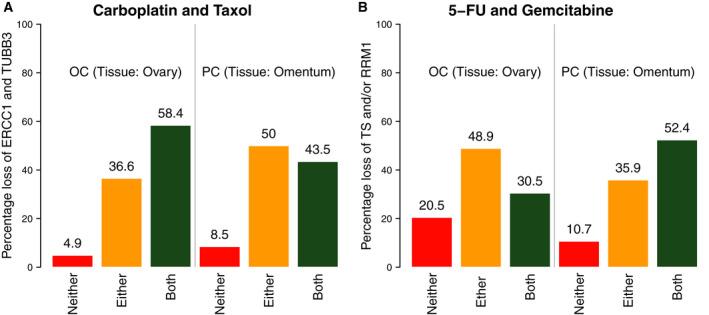
Hypothetical treatment regimens based on combined IHC data (Caris). (A) Percentage of patients with ovarian or peritoneal cancer with IHC‐detected loss of ERCC1 or TUBB3 (orange), loss of both markers (green), or loss of neither (red). Loss of ERCC1 and TUBB3 corresponds with increased likelihood of response to platinum and taxane, respectively. (B) Percentage of patients with ovarian or peritoneal cancer with IHC‐detected loss of TS or RRM1 (orange), loss of both markers (green), or loss of neither (red). Loss of TS and RRM1 corresponds with increased likelihood of response to 5‐FU and gemcitabine, respectively.

With respect to potential targeted therapy approaches, differences in expression were observed for ER (TC: 48.4%; OC: 48.6%; and PC: 55.3%; *P* = 0.049) and PR (TC: 19.6%; OC: 26.4%; and PC: 16.3%; *P* < 0.001). Biomarkers associated with the likelihood of response to classical chemotherapy were also expressed differentially between the two diseases: TOP2A (TC: 81.4%; OC: 79.6%; and PC: 67.5%; *P* < 0.001), RRM1 loss (TC: 72.9%; OC: 74.8%; and PC: 83.3%; *P* = 0.002), and TS loss (TC: 39.1%; OC: 39.2%; and PC: 54.1%; *P* < 0.001) showed significantly different expression levels when tested with precision IHC among the three cancer types. In regard to predictive markers for outcome of chemotherapy regimens, more patients with OC (58.4%) showed combined loss of ERCC1 and TUBB3 as compared to PC (43.5%) using IHC (Figure 4). *Vice versa*, combined loss of TS and RRM1 was found in PC (52.4%) as compared to OC (30.5%).

## 4 Discussion

High‐grade serous cancer are classified into cancers of the ovary, peritoneum, and fallopian tube, depending on the location where the tumor load appears. Despite their different names, these cancers appear similar and are currently treated identically, owing to an absence of biomarkers effectively discriminating between them. One problem is the lack of knowledge regarding disease origin and development. While we have strong evidence to support the notion that the fimbrial end of the fallopian tube is the source of at least a part of HGSC, namely TC, the source of OC and PC is still uncertain. It is, however, unlikely that one single layer of ovarian surface epithelium or one layer of mesothelium could create cancers of exactly the same histological phenotype as the fimbrial end of the fallopian tube. So far, investigative efforts directed toward unraveling their molecular differences remain compromised due to factors such as limited sample collection, difficulties in establishing the primary site from where they were obtained, inaccuracies in cancer diagnoses, difficulties in modeling, and many more. As the first step in unraveling their relationships, we have evaluated the clinicopathological characteristics and discriminatory features of both cancers with regard to their gene and protein expression and genetic mutational load.

Here, we examined by transcriptomic analysis gene patterns for all high‐grade serous adenocarcinomas by (a) histopathologically classified diagnosis and (b) metastatic/sampling site. With respect to the gene expression analysis performed retrospectively incorporating information derived from both clinicopathological characteristics and tumor location, the diagnostic performance of the top genes (*ADH1B* and *FABP4*) revealed a stable discriminatory power between both cancers, thereby suggesting that both diseases follow different genetic pathways. However, PARP inhibitor‐associated genes *BRCA1*, *BRCA2*, and *RAD51C* were equally expressed in OC and PC (data not shown) which was in contrast to blood CA125 levels being significantly elevated in PC. Four publicly available datasets [20, 28, 29, 30] identified *ADH1B* and *FABP4* as markers of residual disease [31]. Tucker *et al*. [31] described a potential relationship between both genes, while *FABP4* was found to be elevated in omental metastases, suggesting genes as candidates for predicting the outcome of neoadjuvant chemotherapy. Here, residual disease and diagnosis independently predicted patient outcome. This indicates that the aggressiveness of the disease is driven by the underlying biology and probably the reason for a more profound and less easily debulkable cancer. In line with this, expression profiling data in matched ovarian cancer tissue samples suggested consideration of more aggressive metastatic states for therapy development [32]. The discrimination between HGSC became even stronger in our data when we compared the sampling site (ovary *versus* omentum) instead of the diagnosis (OC *versus* PC). Both genes are overexpressed in PC and in omental metastases of OC, indicating that they are genetic markers for progressive disease or potentially implicated in the ability of the cancer to metastasize to the omentum. On the one hand, the upregulation of *FABP4* might mechanistically be a consequence of adipocytes located in the omentum or peritoneum promoting homing, migration, and invasion of ovarian or tubal cancer cells to the omentum [33], with no similar support being present at the sites of the ovaries themselves. On the other hand, recent work suggests that *FABP4* increases tumor progression expressed in ovarian cancer cells and is regulated through miR‐409‐3p, triggering metastatic pathways and altering the metabolic state of cancer cells itself [34]. *Vice versa*, *TSPAN8* upregulated in OC and grouped into C3 to C6 (Figure 2C) would be a possible therapeutic target to reduced metastatic disease previously demonstrated in a preclinical setting using TSPAN8‐blocking antibody [35].

More recently, we also demonstrated in another study on post‐translation modifications of proteins a specific expression of *N*‐glycan‐containing LacdiNAc in OC and ovarian tissue [23]. This is in line with another study suggesting LacdiNAc as a tumor‐specific glycan in breast and ovarian cancer cells [36]. Interestingly, the presence of LacdiNAc seems to be associated with elevated gene expression of specific β4‐GalNAc transferases [37, 38] in ovarian cancer tissue and cell lines as compared to the peritoneum. These findings support the concept that different sites of ovarian cancer demonstrate distinct biology, although they might arise from the same cell of origin, namely in the fallopian tube.

The concept of personalized treatment is based upon NGS, CISH, and IHC data on potentially targetable biomarkers that describe an individual molecular footprint of a tumor. We found that the likelihood of response to standard adjuvant chemotherapy is not as favorable for PC as it is for OC. The combined IHC data of ERCC1 and TUBB3, indicating the likelihood of response to therapy with platinum and taxane [39, 40, 41, 42, 43], respectively, were elevated in patients with OC. This indicates that the combination is likely less effective in PC, where in the majority of patients only one of the markers is expressed. While these data support the current standard of care for OC, they also indicate the need to find a better therapy for PC. In fact, 5‐fluorouracil and gemcitabine [44, 45, 46, 47, 48], a chemotherapy combination which is at present not used at all in the adjuvant treatment setting, may have better potential effectiveness for PC. Unfortunately, there is no prospective randomized controlled trial which incorporates a treatment line with 5‐FU and gemcitabine where we could retrospectively validate our findings in high‐grade serous PC. Therefore, we strongly suggest that in the future care should be taken for the treatment selection and variation in the different histological subtypes of OC and PC. Obviously, this is only a hypothesis and a carefully designed prospective clinical trial would be necessary to validate this approach.

## 5 Conclusion

Our study provides further evidence that OC and PC are two distinct diseases, reflected by epidemiological, clinical, and molecular data. While this study incorporates large numbers of patients and a broad spectrum of methods, it has nevertheless the limitation that it cannot compare all HGSC as the numbers of TC were too low to draw any meaningful conclusions. Therefore, this study is limited to the degree of relationship between OC and PC only. This is the first study to clearly demonstrate that OC and PC are distinct entities; however, it remains unclear whether this is due to the location where they develop or with the sampling site. It is possible that both diseases share the same cell of origin from the tube but subsequently develop in distinct ways depending on the tissue into which they metastasize. Alternatively, it is possible that a specific subpopulation of serous tubal intraepithelial cancer cells with a more mesenchymal differentiation might rather trigger the metastasis to omentum or to peritoneum. However, any definite conclusions regarding these theories cannot be drawn from this study. Future clinical considerations incorporating the origin of each cancer type will ultimately lead to an improvement in diagnostic performance and targeted treatment strategies and truly would reflect the spirit of personalized medicine.

## Conflict of interest

The authors declare no conflict of interest.

## Author contributions

FJ and VAH‐S designed research; FJ, RLM, ABK, KR, and AS, collected data, performed experiments, and analyzed data; and FJ, RLM, and VAH‐S wrote the paper.

## Ethical approval

Ethical approval for this study was granted by the Ethikkommission Nordwest‐ und Zentralschweiz (EKNZ: 2017‐01900). The study was performed in accordance with the Declaration of Helsinki.

## Supporting information


**Table S1.** Summary of ranked genes identified by diagnosis comparing PC versus OC and tissue sampling site.
**Table S2.** Differential Gene expression comparing PC versus OC in regards to diagnosis.
**Table S3.** Differential Gene expression comparing sampling sites.
**Table S4.** Descriptive statistics obtained from NGS, FISH/CISH and IHC in a cohort of serous adenocarcinomas of the fallopian tube, ovary, and peritoneum.

## Data Availability

All data are publicly available or part of this study. Additional data will be made available to the scientific community for nonprofit use upon request.
